# Giant Strain and Induced Ferroelectricity in Amorphous BaTiO_3_ Films under Poling

**DOI:** 10.3390/ma10091107

**Published:** 2017-09-20

**Authors:** Pegah Mirzadeh Vaghefi, Ali Baghizadeh, Armando A. C. S. Lourenço, Vitor S. Amaral, Andre L. Kholkin

**Affiliations:** 1 Department of Physics & CICECO-Aveiro Institute of Materials, University of Aveiro, 3810-193 Aveiro, Portugal; Alourenco@ua.pt (A.A.C.S.L.); vamaral@ua.pt (V.S.A.); kholkin@ua.pt or kholkin@urfu.ru (A.L.K.); 2 Department of Materials and Ceramics Engineering & CICECO-Aveiro Institute of Materials, University of Aveiro, 3810-193 Aveiro, Portugal; ali.baghizhadeh@ua.pt; 3 School of Natural Sciences and Mathematics, Ural Federal University, 620000 Ekaterinburg, Russia

**Keywords:** BaTiO_3_, ferroelectricity, piezoresponse force microscopy, self-assembly, poling

## Abstract

We report an effect of giant surface modification of a 5.6 nm thick BaTiO_3_ film grown on Si (100) substrate under poling by conductive tip of a scanning probe microscope (SPM). The surface can be locally elevated by about 9 nm under −20 V applied during scanning, resulting in the maximum strain of 160%. The threshold voltage for the surface modification is about 12 V. The modified topography is stable enough with time and slowly decays after poling with the rate ~0.02 nm/min. Strong vertical piezoresponse after poling is observed, too. Combined measurements by SPM and piezoresponse force microscopy (PFM) prove that the poled material develops high ferroelectric polarization that cannot be switched back even under an oppositely oriented electric field. The topography modification is hypothesized to be due to a strong Joule heating and concomitant interface reaction between underlying Si and BaTiO_3_. The top layer is supposed to become ferroelectric as a result of local crystallization of amorphous BaTiO_3_. This work opens up new possibilities to form nanoscale ferroelectric structures useful for various applications.

## 1. Introduction

The power of nanotechnology is that it offers novel high resolution nanopatterning techniques to overcome the restrictions of conventional lithography [1,2]. With that purpose, several lithographic methods have been developed in the past, including those based on scanning probe microscopy (SPM) (scanning probe lithography [3], nano stencil lithography [4,5], dip-pen nanolithography [6,7]) and methods based on stamps or masks (soft-lithography [8], nanoimprinting [9], nano sphere lithography [10,11,12], and unconventional wet lithography [13,14]), nanoimprint lithography (NIL), [15,16] and electrochemical lithography [17]. These methods offer important advantages, such as high efficiency (soft and nano sphere lithography), suitability for large areas (soft, unconventional lithography and nanoimprinting), a few nanometers resolution (nanoimprinting, scanning probe lithography, nanostencil, and dip-pen nanolithography), high versatility (soft unconventional lithography and nanoimprinting), and direct processability of solutions (soft and unconventional lithography).

In the recent years, much attention has been paid to the ferroelectric lithography that relies on polarization reversal by the tip of a piezoresponse force microscope (PFM) with the following exposure to polarization sensitive media [18]. These methods typically use local polarization reversal accompanied by reversible strain that does not exceed 1% even if a very high electric field is applied [19,20]. Morphological changes due to the application of an external electrical bias, i.e., electric poling treatment have been previously observed and attributed to the interaction between conductive probe and the trapped charges injected into the film during poling process in polymers [19] and PZT/LSMO thin films [20]. Large changes in the morphology were observed in a-Si films due to SPM-induced crystallization and expected increase of the materials density [21]. In oxide ferroelectrics, these effects have not been seen so far.

In this work, we report the observation of a giant poling effect on the topography of amorphous BaTiO_3_ ultrathin films deposited on Si substrates by RF (radio frequency) magnetron sputtering. Along with the topography modification we found the appearance of a strong vertical piezoresponse and polarization hysteresis that hint to the possible ferroelectricity of initially paraelectric films. This work illustrates a novel approach to nanoscale lithography enabling the creation of ferroelectric nanostructures controlled by the voltage applied to the SPM tip.

## 2. Experimental

BaTiO_3_ films were deposited on (100) Si substrates, using RF magnetron sputtering technique from stoichiometric target. Prior to deposition the substrates were chemically cleaned with hydrofluoric acid-based solution (deionized water + methanol + 40% fluoric acid) for 120 s to remove SiO_2_ layer and then were rinsed with deionized water. The films were grown in 1:10 oxygen to argon atmosphere at 630 °C and RF power of 25 W. The thickness of the films was about 5.6 nm. The structure of the produced films was checked by X-ray diffraction (XRD) and scanning transmission electron microscopy (STEM) using a 200 kV Hitachi HD2700 system.

We used a commercial scanning probe microscope (NTEGRA PRIMA, NT-MDT Spectral Instruments, Moscow, Russia) to investigate both the surface morphology and local piezoelectric properties of the films before and after poling. The samples were electrically poled in the PFM mode by applying voltage pulses between the conductive tip and Si substrate (−20 V < *V* < +20 V) during scanning. We used Si cantilevers with Pt/Cr tips from Nanosensors Co., Neuchâtel, Switzerland. The tip height was 10–15 μm and resonance frequency and force constant were 204–497 kHz and 10–130 N/m, respectively. Poling was done while scanning the surface with the tip velocity of ~4 μm/s.

## 3. Results and Discussion

**Figure 1:**
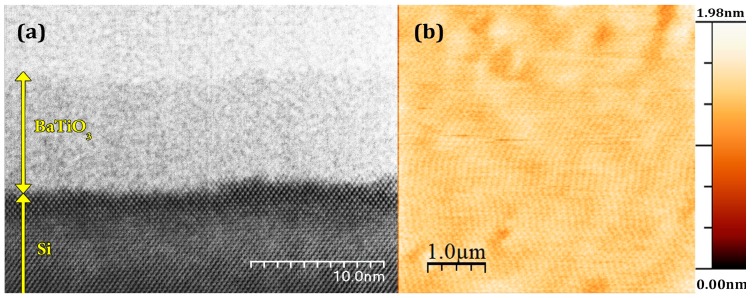
(**a**) Bright field STEM image of a 5.6 nm-thick BaTiO_3_ film on Si substrate and (**b**) topography of the film before poling (RMS roughness 0.18 nm).

**Figure 2:**
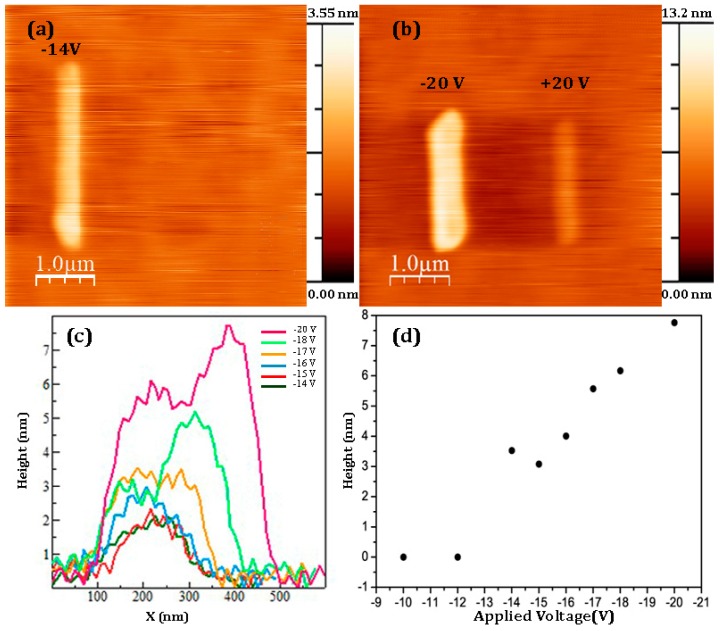
Topography of BaTiO_3_ thin film after application of ±14 V (**a**) and ±20 V; (**b**) during scanning of the area 0.2 × 2 μm^2^; (**c**) Comparison of the topography cross-sections of the areas poled with different voltages; (**d**) Average height of the poled areas vs. applied negative voltage.

**Figure 3:**
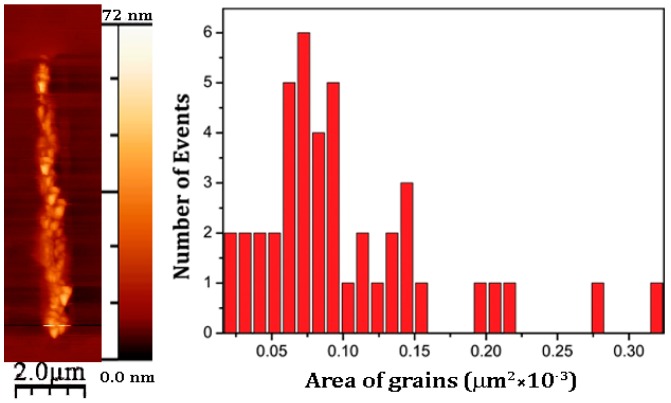
Topography at high magnification after application of −20 V applied to the surface during scanning of the area 0.2 × 2 μm^2^ (**left**) and grain size distribution of the crystallized BaTiO_3_ film after poling (**right**).

Cross-sectional STEM analysis of the as deposited BaTiO_3_ film is shown in Figure 1a. It is clear that the film is amorphous with well-defined interface between BaTiO_3_ and underlying substrate. The thickness of the films was about 5.6 nm as measured by cross-sectional analysis. The absence of SiO_2_ layer is expected because the substrate was rigorously cleaned before deposition. Representative topography of unpoled BaTiO_3_ thin film is shown in Figure 1b. The surface is very smooth with the root-mean-square (RMS) roughness of about 0.18 nm. No piezoresponse was observed by PFM because the perovskite structure was not formed during the low-temperature deposition process. The film was poled during scanning of 20 × 0.2 μm^2^ areas by the application of the dc bias voltage *V_app_* in the range −20 V < *V_app_* < +20 V at 24.2 lines per minute. It can be clearly seen that after poling with negative voltage *V_app_* = −14 V (*E* = 25.5 MV·cm^−1^), a bump of about 3.5 nm in height is visible across the poled area (Figure 2a). By increasing the applied voltage to *V_app_* =−20 V the height of the poled area was increased to 7.2 nm, i.e., the maximum irreversible strain was about 128% (Figure 2b). Strong strain asymmetry was observed: Even under the highest voltage (+20 V), the modified area was much lower under positive voltage applied to the tip. It has to be noted that the modified area is wider than the poled region, indicating that thermal heating or mechanical stress (rather than electric field itself) can be involved in the formation of nano islands. In addition, the topography of the poled area is much less uniform than the initial surface as shown in the topography cross-sections (Figure 2c) and clear grains of triangular shape are present in contrast to the smooth surface of pristine (unpoled) BaTiO_3_ films (Figure 3).

Average height of the poled area vs. applied negative voltage is shown in Figure 2d. It can be seen that the deformation starts at about −12 V and then almost linearly increases up to −20 V. After the analysis of grain size distribution shown in Figure 3, we infer that the average grain size of the poled area is about 100 nm, which is consistent with the grain size of (111) oriented crystalline BaTiO_3_ films prepared by metalorganic chemical vapor deposition [22]. Note that, in order to crystallize the films, substrate should be heated to about 800 °C. Interestingly, triangular shape of the grain may also come from the (111) orientation of BaTiO_3_, which is the direction of highest piezoelectric response [23].

**Figure 4:**
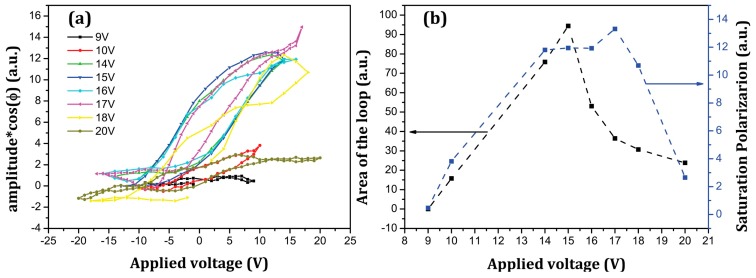
PFM hysteresis loops measured under different voltages (**a**) and maximum voltage dependence of the area of the loop and maximum piezoresponse (**b**).

We also measured piezoelectric response and its hysteresis directly on the elevated areas after poling (Figure 4a,b).

**Figure 5:**
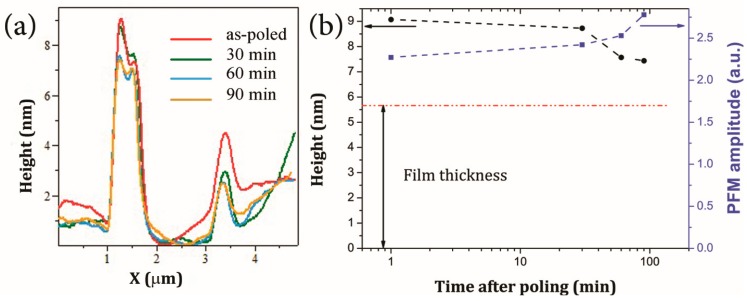
(**a**) Evolution of the topography cross-section with time; (**b**) average height and PFM amplitude of the peak over measuring time created by the +20 V poled area. The red line shows the initial thickness of the film.

When the maximum applied voltage is small (~9 V) no piezoresponse and its hysteresis are visible. With increasing bias voltage, the loops begin to open and piezoresponse starts to grow, reaching the maximum value at about 17 V. After that, a significant drop of the piezoresponse occurs and hysteresis shrinks hinting to a possible degradation of the created ferroelectric nanostructure. The lower threshold for the disappearance of the ferroelectricity as compared to topography (Figure 2d) could result from different measurement mode. In case of raster scanning, the threshold is higher because the surface experiences the voltage application at much shorter time as compared to the point poling during hysteresis measurements. All hysteresis curves demonstrate negligible switching when the opposite voltage is applied to the tip. Thus, it can be confirmed that a very stable polarization state is created under poling similar to that formed during fatigue [24]. The height of the structure does not significantly change with time after poling. Figure 5 shows the evolution of the created nanostructures with time. While the height slowly decays after poling with a rate of ~0.02 nm/min, the apparent piezoresponse increases by about 20% after ~100 min.

**Figure 6:**
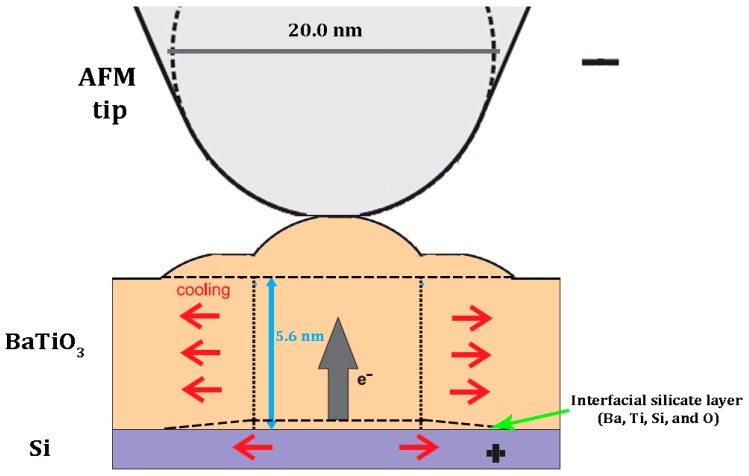
Schematic of the formation of modified structure of amorphous BaTiO_3_ film under poling (not to sale).

One of the possible explanations for surface modification can be a material transfer from the tip [20]. However, this mechanism can be ruled out, since no continuous degradation of image quality is observed after several scans, whereas there is a significant topographical change. Another mechanism that can be involved is due to crystallization of the amorphous BaTiO_3_ thin film material under high current flowing at the voltage exceeding 12 V. This is analogous to the observed effect of crystallization of amorphous Si under the voltage applied to the SPM tip [21]. The Joule effect can naturally explain the asymmetry of the effect in respect to the sign of applied voltage. Indeed, the current vs. voltage characteristics of metal-a-BaTiO_3_-Si structures are often asymmetric due to the formation of Schottky barriers at the interfaces [25]. However, in this case, a dip in topography is expected because of the lower density of the amorphous materials as compared to the crystallized one. Another difficulty in the explanation of possible (111) orientation of crystallized BaTiO_3_ is because pure Si (100) surface promotes *a*-orientation of BaTiO_3_ [23]. In our case, both difficulties can be overcome if we consider a surface reaction at the BaTiO_3_/Si interface under the high temperature caused by the applied voltage above 12 V. Crystallization of BaTiO_3_ at the surface can be accompanied by the formation of a nm thick interfacial silicate layer composed of Ba, Ti, Si, and O. This phase may correspond to fresnoite, Ba_2_TiSi_2_O_8_, [26] and the thickness of entire slab may significantly increase due to the lower density of fresnoite, 4.43 g/cm^3^, about 50% smaller than that of BaTiO_3_ (6 g/cm^3^). Though the density of crystalline BaTiO_3_ is higher than the amorphous one, porous grain structure shown in Figure 3a may lead to the decrease of average density and thus to the increase of the height, opposite to the effect observed in amorphous Si [21]. The schematic of the cross-section formed under the PFM tip is shown in Figure 6. Using Hertz elasticity theory and the known force applied to the cantilever, we can determine the value of contact diameter of about 7 nm. We assume that the Joule heat from the high current appearing between the tip and the Si is enough to crystallize the top area just under the tip. The field distribution is asymmetric because the contact diameter is only a bit larger than the film thickness. Therefore, the current density is higher under the tip and this part of the film crystallizes under the voltage application. It should be noted that poling is very efficient because the voltage is applied above the Curie point. The charges created by the voltage are injected into the film and trapped at the interface thus creating a strong polarization offset seen in Figure 4a. This field still aligns dipoles in BaTiO_3_ even after the voltage is switched off (Figure 5b). The lower part of BaTiO_3_ film apparently experiences lower temperature increase and forms the interface layer (possibly fresnoite) due to strong diffusion of Si into amorphous BaTiO_3_. Thus, a very stable composite structure is formed consisting of crystalline BaTiO_3_ on the top and silicide interface layer on the bottom. The (111) orientation of BaTiO_3_ can be a result of the seeding effect of fresnoite that forms earlier. Further local structural investigation is needed to corroborate this model. The formation of ferroelectric nano-islands is important for the memory applications [27]. In principle, the size of the ferroelectric nano-islands can be as small as the contact area provided by the tip. Short voltage pulses are applied to crystallize BaTiO_3_ locally with heating up a larger area. However, the intrinsic imprint, i.e., the tendency of ferroelectric to be in one (preferred) polarization state as seen in Figure 4a can be an obstacle. In this case, the application area can be polarization lithography as described in detail in the comprehensive review by Kalinin et al. [18].

## 4. Conclusions

A systematic study of topography modification of ferroelectric BaTiO_3_ films on Si under poling was performed. The surface was locally elevated by about 9 nm, due to −20 V applied voltage by scanning technique, which results in 160% strain in poled region. The threshold voltage for modifying the surface was about 12 V. Also, the stability of modified topography with time was examined, showing a slow decay after poling at a rate of 0.02 nm/min. Combined measurements by SPM and piezoresponse force microscopy showed that the poled material develops high polarization that cannot be switched back, even under a high electric field. Strong vertical piezoresponse was observed, which increased with time after poling. The topography modification is thought to be due to strong Joule heating and concomitant interface reaction between underlying Si and BaTiO_3_ forming silicide. The top layer of amorphous BaTiO_3_ film becomes ferroelectric because of local crystallization by Joule heating. This work opens up new opportunities to form nanoscale ferroelectric structures by SPM technique, useful for various applications, e.g., polarization lithography.
